# G Protein-Coupled Receptor Signaling in Stem Cells and Cancer

**DOI:** 10.3390/ijms17050707

**Published:** 2016-05-11

**Authors:** Jennifer R. Lynch, Jenny Yingzi Wang

**Affiliations:** 1 Cancer and Stem Cell Biology Group, Children’s Cancer Institute, Lowy Cancer Research Centre, University of New South Wales, Sydney, NSW 2052, Australia; JLynch@ccia.org.au; 2 Centre for Childhood Cancer Research, Faculty of Medicine, University of New South Wales, Sydney, NSW 2052, Australia

**Keywords:** GPCR, G proteins, signaling, stem cells, cancer stem cells, therapy

## Abstract

G protein-coupled receptors (GPCRs) are a large superfamily of cell-surface signaling proteins that bind extracellular ligands and transduce signals into cells via heterotrimeric G proteins. GPCRs are highly tractable drug targets. Aberrant expression of GPCRs and G proteins has been observed in various cancers and their importance in cancer stem cells has begun to be appreciated. We have recently reported essential roles for G protein-coupled receptor 84 (GPR84) and G protein subunit Gα_q_ in the maintenance of cancer stem cells in acute myeloid leukemia. This review will discuss how GPCRs and G proteins regulate stem cells with a focus on cancer stem cells, as well as their implications for the development of novel targeted cancer therapies.

## 1. Introduction

Stem cells are the cells that have the ability to make identical copies of themselves for the lifetime of the organism (self-renewal), and can also divide to generate progenitor or precursor cells that then differentiate into all cell types of a mature tissue (differentiation) [1]. In general, stem cells are classified into two categories depending on the plasticity of their pluripotent and differentiation potential. Embryonic stem cells (ESC) are derived from the inner cell mass of the blastocyst stage of the embryo and are pluripotent, having the ability to generate any tissue in the body but unable to generate a complete individual on their own [2,3,4]. Adult stem cells, on the other hand, are multipotent, exhibiting a more restricted differentiation capacity, and only persist in specific niches throughout postnatal life [5,6]. Certain adult stem cells such as hematopoietic, mesenchymal and neural stem cells can differentiate into multiple lineages, while others, such as endothelial and corneal stem cells are significantly lineage restricted and only possess the ability to differentiate into one cell type [7]. A delicate balance between stem cell self-renewal and differentiation defines major organ development that results in ordered layers of functional differentiated cells and residual stem cells responsible for renewal and repair [8,9].

Several studies have now confirmed the central role played by GPCRs in embryonic development and stem cell maintenance [10,11,12]. Dysregulation of these fundamental biological processes can have detrimental consequences including malignant transformation [13,14]. Increasing evidence has now demonstrated the malignant transformation of normal stem cells into cancer stem cells (CSCs) via the accumulation of various genetic modifications [15]. CSCs appear to hijack signaling pathways (e.g., GPCR) and mechanisms that regulate normal stem cells, adapting the ability to self-renew and thereby regenerating tumors after anti-cancer treatment [16,17]. While current treatment regimens kill the bulk of tumor cells, they ultimately fail to induce durable clinical responses because CSCs develop treatment resistance over time due to their quiescent nature [18,19,20,21]. Understanding the signaling pathways in normal and malignant stem cells will facilitate the use of normal stem cells for regenerative medicine and the development of new therapies to target CSCs.

## 2. The Diversity of GPCR Signaling Mechanisms

**Figure 1:**
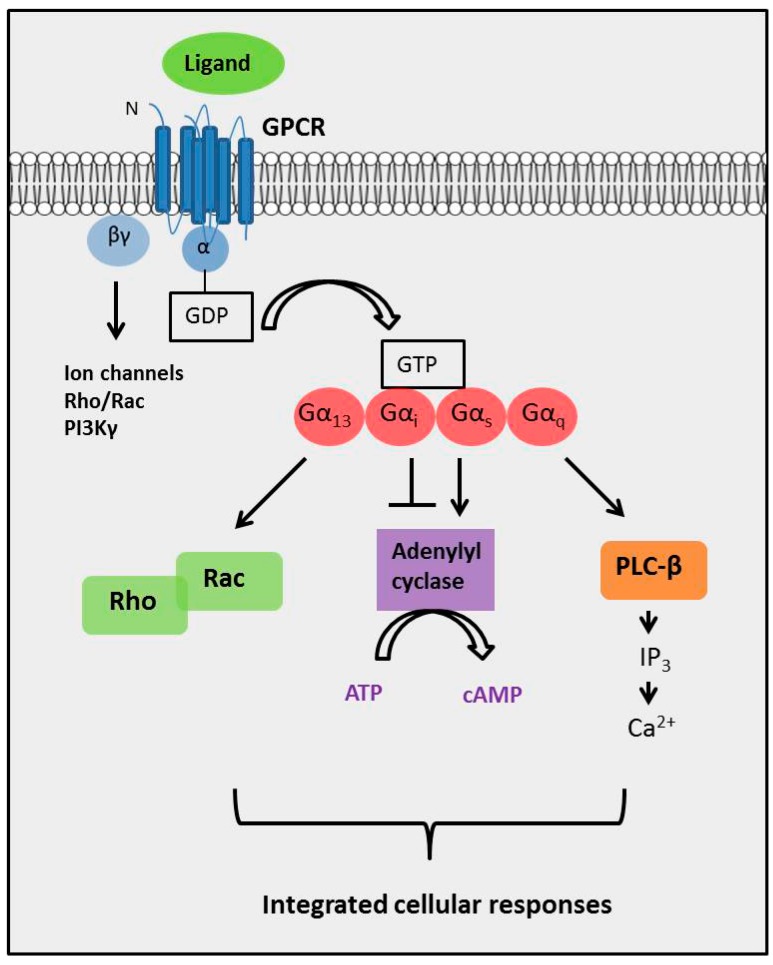
GPCRs signal through heterotrimeric G proteins. GPCRs transmit extracellular signals across the plasma membrane to intracellular effectors via heterotrimeric G proteins [22]. G proteins belong to the GTPase family and are composed of three subunits, α, β and γ, in which the β and γ subunits form a stable dimeric complex, the βγ-subunit [44]. Upon agonist stimulation, the GPCR undergoes rapid conformational changes that expose intracellular sites that interact with and activate G proteins [45]. This catalyzes the dissociation of GDP bound to the Gα subunit and its replacement with GTP, in turn leading to the dissociation of Gα from the βγ-subunit [46]. Both Gα-GTP and Gβγ-subunit complexes are then freely available to activate downstream effectors [28,47,48]. Gα subunits have been classified into four families: Gα_s_, Gα_i/o_, Gα_q_ and Gα_12/13._ The Gα subunits activate multiple downstream effectors ultimately leading to alterations in gene expression allowing the cell to adapt to external stimuli. The Gα_s_ and Gα_i_ family subunits regulate the activity of adenylate cyclase, thereby altering cAMP levels [25]. Gα_13_ primarily activates the Rho family of GTPases and Gα_q_ stimulates phospholipase-Cβ (PLC-β) leading to mobilization of intracellular Ca^2+^ [26,27]. Abbreviations: GDP, guanosine diphosphate; GTP, guanosine triphosphate; cAMP, cyclic adenosine monophosphate; Rho, Ras homolog family; Rac, Ras related small GTPase protein; IP_3_, inositol triphosphate; ↓: Signaling activation; ⊥: signaling inhibition.

GPCRs are versatile signaling molecules that modulate the activities of diverse intracellular signaling via G proteins [22]. G proteins consist of Gα, Gβ and Gγ subunits, and Gα subunits have been classified into four subfamilies: Gα_s_, Gα_i/o_, Gα_q/11_ and Gα_12/13_, based on structural and functional similarities [23]. Each Gα family can relay the GPCR signals to multiple downstream effectors, consequently triggering different signaling pathways [24]. The Gα_s_ and Gα_i/o_ families function to activate or inhibit the activity of adenylate cyclase with a consequential increase or decrease in cyclic AMP (cAMP) production [25]. Members of the Gα_q/11_ family activates phospholipase-Cβ (PLCβ) ultimately leading to intracellular Ca^2+^ mobilization from the edoplasmic reticulum [26]. In addition, the Gα_12/13_ family is involved in activation of the Rho family of small GTPases [27]. These downstream effectors of G proteins subsequently trigger various intracellular signaling pathways that modulate diverse cellular functions (Figure 1) [26,28]. The major known targets of downstream G protein signaling include ion channels, calcium-sensitive enzymes, and kinases such as cAMP-dependent kinase (PKA), protein kinase X and calcium-calmodulin regulated kinases (CAMKs). Many of these kinases play contributing roles to cancer development and progression [29,30,31].

Following activation of GPCRs, rapid attenuation or desensitization of receptor responsiveness is necessary to prevent uncontrolled signaling. Desensitization is initiated by phosphorylation of the receptor by GPCR kinases [32,33] followed by uncoupling of GPCR-G protein interactions mediated by members of the β-arrestin protein family [34,35]. In addition to terminating G protein signaling, β-arrestins also play a role in promoting GPCR signaling by internalizing the receptor and acting as a molecular scaffold to recruit signaling proteins. In this way β-arrestins are capable of initiating G protein independent GPCR signaling cascades. This again challenges the traditional concept of GPCR activation involving a single ligand and receptor pair. It is now apparent that various ligands can activate a single GPCR to stabilize specific ligand-receptor conformations that promote unique signaling properties. Another critical point in the negative regulation of GPCR signaling is the deactivation of Gproteins by GTP hydrolysis, which is enhanced by Regulator of G protein Signaling Proteins (RGS). RGS proteins are capable of accelerating GTPase activity up to 1000-fold [36] and can also serve as effector agonists by competitively binding activated Gα-subunits [37,38] or promote rapid cycling of Gα-subunits between active and inactive states thereby serving as kinetic scaffolds [39]. Complex cross-talk between ligands, receptors, G proteins, second messengers, and accessory proteins facilitates the diverse range of GPCR signaling as it is recognized today.

GPCR signaling is highly diverse and the engagement of different G proteins and the strength or duration of signaling differs not only between GPCRs, but also depending on the ligand and cellular environmental context of any given GPCR. Some GPCRs, such as sphingosine-1-phosphate receptor 1 (S1P1), exclusively couple to one G protein, whereas other GPCRs, such as lysophosphatidic acid receptors (LPA), can couple to multiple G proteins triggering diverse downstream signaling cascades [40,41]. Aberrant activation of GPCR signaling triggered by high-affinity ligands (e.g., LPA and S1P1) leads to malignant transformation, proliferation, metastasis and drug resistance [42,43].

### 2.1. GPCR-Mediated Regulation of Stem Cell Properties

The role of GPCR signaling in stem cell function, although undoubtedly important, has not been fully elucidated. Several lines of evidence are suggestive of a critical role, for instance many of the signaling cascades activated by GPCR signaling directly regulate, or are synergistic with, pathways that regulate ESC pluripotency and differentiation [49]. General roles for G proteins in regulating pluripotency have also been described. Gα_s_ signaling has been shown to promote proliferation and pluripotency in self-renewing and differentiating mouse ESCs [11]. Signaling mediated by Gα_i_ proteins is demonstrated to affect the morphology and organization of human induced pluripotent stem cells (iPSC) [10]. Dramatic changes in the expression levels of GPCRs in distinct stages of stem cell differentiation further implicate their involvement in stem cell function [50]. Comprehensive qPCR analysis of more than 350 GPCR genes between three stages of *in vitro* neural differentiation (*i.e.*, pluripotent human ESCs, multipotent neural progenitors, and differentiated neurons) has revealed striking differences in GPCR expression within the different cell populations [50,51].

The GPCR superfamily is divided into five sub-families, including glutamate, frizzled, adhesion, rhodopsin and secretin. The specific roles of GPCRs from two of these families (*i.e.*, frizzled and rhodospin) will be discussed in this review exemplifying GPCR-mediated regulation of normal and malignant stem cells.

### 2.2. Wnt-Activated Fzd Signaling

The frizzled (Fzd) family of GPCRs is activated by the Wnt family of lipoglycoproteins [52]. Wnt signaling plays a critical role during development and Wnt ligands are known to regulate the maintenance of numerous stem cell populations in both developing and adult organisms [53]. Although Fzds have normal GPCR topology, their lack of sequence similarity leads to debate regarding their classification as GPCRs [54]. Nevertheless, compelling experimental observations show that heterotrimeric G proteins play a crucial role in Wnt signaling, specifically given that Wnt signaling activation could be abrogated in human ESCs by a Gα_i/o_ protein inhibitor (pertussis toxin) [55,56]. Furthermore, Gαo has been shown to be essential for Wnt activation in *Drosophila* [57]. As such, the Fzd family is listed by the International Union of Pharmacology as a novel and separate family of GPCRs termed “Class Frizzled” [58].

Nineteen Wnt proteins serve as the primary endogenous agonists for 10 Fzd receptors encoded in the human genome [59]. There is apparent specificity between individual Fzds and their ligands with Wnt3a-Fzd1, Wnt5a-Fzd7 and Wnt7-Fzd6 being identified as highly efficient Wnt-Fzd pairs [60,61]. Three main pathways in Wnt-activated Fzd signaling include: Fzd/Ca^2+^ pathway, Fzd/planar cell polarity (PCP) pathway and Fzd/β-catenin pathway. Agonist stimulation of the Fzd/Ca^2+^ pathway leads to elevated intracellular Ca^2+^ levels in a G protein-dependent manner that activates calcium-dependent protein kinase c (PKC) and Ca^2+^/calmodulin-dependent protein kinase [62,63]. The Fzd/PCP pathway tranduces via Dishevelled (Dvl) to small Rho GTPases and their effectors Rho-associated coiled-coil containing protein kinase (ROCK) and the c-Jun-N-terminal kinase/c-Jun/AP-1 pathway [64]. Agonist stimulation in the Fzd/β-catenin pathway activates the phosphoprotein Dvl, leading to inhibition of the destruction complex composed of adenomatosis polyposis coli protein (APC) and Axin. β-catenin then translocates from the cytoplasm to the nucleus, where it cooperates with the T-cell factor/lymphoid enhancer factor (Tcf/Lef) transcription factors to modify transcription of a set of Wnt target genes [53]. The Wnt/Fzd pathways have been classified as regulators of cell fate determination and control cell movement and tissue polarity, respectively [49].

Fzd receptors play an important role in mammalian development and stem cell self-renewal. The expression of Fzd5, 7 and 10 has been found in the gastrulating embryos of mice and is implicated in neural induction [65]. Evidence from knockout mouse studies suggests that Fzd4, 5 and 9 are important for central nervous system development and self-renewal of B cell populations [66,67,68]. Various studies suggest that Wnt3a inhibitor or GSK-3 inhibitor (6-bromoindirubin-3’-oxime) maintains pluripotency in human ESCs [69]. In particular, the mRNA levels of the Wnt receptor Fzd7 are found to be 200-fold higher in human ESCs compared to differentiated cell types, and Fzd7 knockdown induces significant morphological changes in ESC colonies with concomitant loss of the pluripotency gene octamer-binding protein 4 (*Oct-4*) [70]. In contrast, some studies have reported a pro-differentiation role for Wnt/Fzd signaling [71,72,73] suggesting a cellular context-dependent effect of Wnt signaling on stem cell self-renewal.

Wnt/β-catenin signaling is evolutionarily conserved and plays critical roles in development and disease [74]. Inappropriate pathway activation produces uncontrolled cell growth leading to cancer, and aberrant Wnt signaling has been implicated in different types of cancer, including hepatcellular carcinomas, ovarian carcinomas, leukemia, prostate cancers, colon cancers and melanoma [75]. Indeed, the extent to which carcinomas rely on Wnt signaling to drive their development and progression is exemplified by the observation that approximately 90% of colon cancer and 50% of breast cancer cases are associated with hyperactivation of Wnt signaling [76,77].

Dysregulation of Wnt/β-catenin signaling occurs in multiple types of CSCs and increasing evidence has demonstrated a crucial role for Wnt/β-catenin signaling in the self-renewal and malignant behavior of CSCs [78,79,80,81]. We as well as others have previously demonstrated that aberrant activation of Wnt/β-catenin signaling contributes to the transformation of hematopoietic stem cells (HSCs) into leukemic stem cells (LSCs) [78,82]. Furthermore, our laboratory has recently identified GPR84 as a novel regulator of β-catenin signaling in LSCs [83]. GPR84 overexpression induces the activation of β-catenin transcriptional co-factors Tcf7l2 and c-Fos, as well as a gene set associated with Wnt signaling [69]. Our functional study shows that GPR84 depletion impairs LSC function and inhibits the development of an aggressive and drug-resistant subtype of acute myeloid leukemia (AML) [83]. Importantly, the GPR84-deficient phenotype is β-catenin dependent as re-expression of active β-catenin is capable of rescuing the deficiency [69]. In addition, levels of GPR84 expression are significantly upregulated in human and mouse AML LSCs compared to normal HSCs [83], providing a therapeutic window to selectively target LSCs while sparing normal HSCs. These studies demonstrate a strong rationale for inhibiting GPCR/β-catenin signaling as a novel therapeutic strategy to target drug-resistant malignant stem cells in cancer.

In support of this therapeutic rationale, we have recently shown an essential role for G protein subunit Gα_q_ in the maintenance of AML LSCs [84]. By using both shRNA-mediated silencing and pharmacological inhibition, our study shows that Gα_q_ regulates LSC growth and survival *in vitro* and *in vivo*, and controls β-catenin activity. Using a commercially available Gα_q_ inhibitor, GP-antagonist 2A, our data indicates that *ex vivo* pre-treatment of LSCs with the antagonist impairs their proliferative capacity in mouse bone marrow and prolongs mouse survival [84]. Therefore, further investigations into the therapeutic applicability of GP-antagonist 2A for the treatment of AML are significantly warranted. In addition, we have shown that inhibiting Gα_q_ expression leads to suppression of mitochondrial complex 1 subunits (*i.e.*, Nd2, Nd4l, Nd5) and consequent disruptions in mitochondrial function and energy metabolism in leukemic cells [84], providing a mechanism linking mitochondrial dysfunction with leukemogenesis via Gα_q_ signaling activation. Thus, targeting β-catenin signaling and energy metabolism by blocking Gα_q_ signaling could represent a novel therapeutic approach to reduce leukemogenesis in aggressive AML.

### 2.3. Rhodospin Class of GPCRs

The rhodopsin class is by far the largest GPCR family, comprising almost 85% of GPCRs. The leucine-rich repeat-containing (Lgr) proteins are a distinct subset of evolutionarily conserved rhodopsin GPCRs containing a large extracellular domain with multiple leucine-rich repeats [85]. The Lgr family member Lgr5 is a known stem cell marker in certain types of tissue [86,87]. *In vivo* lineage tracing experiments using a heritable-inducible lacZ reporter gene introduced into Lgr5-expressing cells has shown that Lgr5 is a marker of adult intestinal stem cells. Further examination of Lgr5 expression patterns in mice has identified discrete populations of Lgr5-expressing cells in organs including skin, stomach, mammary gland, tongue, kidney and endometrium, indicating that Lgr5 may function as a universal epithelial stem cell marker [86,88,89,90,91].

**Figure 2:**
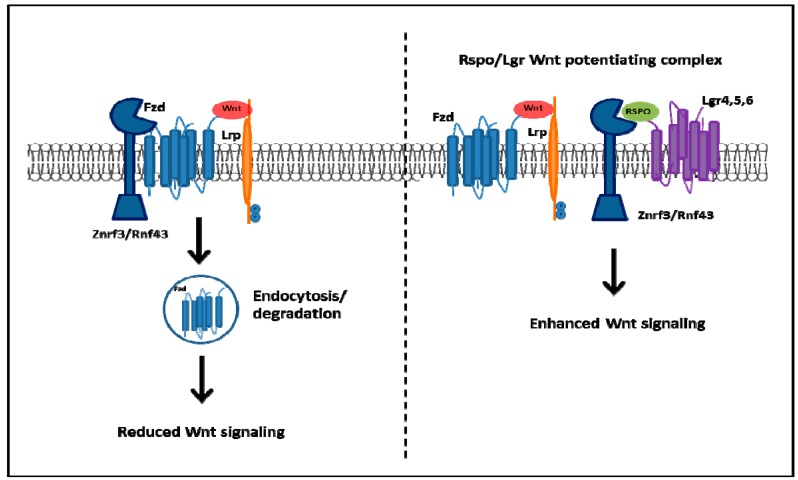
The intensity of Wnt signaling is enhanced by the formation of the Rspo/Lgr-Wnt potentiating complex at the cell membrane. The Wnt-activated ubiquitin ligases, Rnf43 and Znrf3, function in a negative feedback circuitry to control the intensity of Wnt signaling activation. Rnf43/Znrf3 binds to Fzd receptors leading to polyubiquitination, endocytosis and destruction by lysomes [87]. In the presence of Rspo Rnf43/Znrf3 is neutralized facilitating the accumulation of Fzd receptors at the plasma membrane and enhances the intensity of Wnt signal strength [85].

Epithelial homeostasis in the adult intestine is regulated by several signaling pathways and key among these is the Wnt signaling pathway [92]. Hyperactivation of the Wnt signaling pathway is associated with transformation of the intestinal epithelium [93]. Lgr5 has been identified as a Wnt target gene and overexpression of Lgr5 antagonizes Wnt signaling [94,95,96]. The exact mechanism remains unknown but the potential outcome of Lgr5 antagonism would result in β-catenin phosphorylation and targeting for degradation [76]. In addition, overexpression of Lgr5 in colon cancer and HEK293 cells decreases cell motility and stimulates cell-cell adhesion [97]. R-spondin proteins (Rspo1-4) have been identified as ligands of the Lgr family [98]. The inhibitory effect of Lgr5 appears to be abolished in the presence of Rspo [76], and one potential model for potentiation of Wnt involves direct interaction and formation of a Wnt-potentiating complex, Rspo/Lgr5/Wnt/Fzd, at the plasma membrane [94]. Two highly homologous Wnt target genes, Rnf43 and Znrf3, also play a role in the complex regulation of Wnt signaling at the receptor level. Both Rnf43 and Znrf3 are ubiquitin ligases found specifically in Lgr5 crypt stem cells and enriched in colon cancer [99,100,101]. These ubiquitin ligases mediate multiubiquitination of lysines in the cytoplasmic transmembrane domains of Fzds that results in rapid endocytosis of Wnt receptors and their destruction by lysosomes. Loss of Rnf43 and Znrf3 expression results in hyperresponsiveness to Wnt signals leading to the formation of abnormal adenomas consisting entirely of Lgr5 stem cells [100]. Since Rnf43 and Znrf3 are encoded by Wnt target genes, this represents an intricate negative feedback loop controlling Wnt receptor expression [81]. Furthermore, it has been demonstrated that the Rnf43/Znrf3-mediated membrane clearance of Wnt receptors can be reversed by R-spondin [101], and thus Rspo-Lgr complexes neutralize Rnf43/Znrf3 to allow persistence of Fzds receptors and boosting Wnt signal strength (Figure 2).

A recent study by Baker *et al.* has demonstrated the role of Lgr5-expressing cells in the development of colon cancer. By applying *in situ* hybridization (ISH) to a panel of human normal colon, adenoma and carcinoma samples, significant increase in levels of Lgr5 mRNA is observed in all serrated lesions that is accompanied by expansion of proliferative and invasive compartments, suggesting that Lgr5 may support invasion and metastasis [102]. Within the colonic crypts, many cells are endowed with stem cell potential but only a small percentage of the total Lgr5-expressing cells actually functions as stem cells at any one time [103]. Within the malignant adenomatous crypts, however, dysregulation of the processes that govern normal stem cell maintenance results in an elevated number of functional stem cells [104]. Deducing the connection between Wnt signaling, Lgr5 signaling and cancer cell migration will improve our understanding of the role for GPCR signaling in migration of normal and malignant stem cells.

Interestingly, Lgr4 and Lgr6, two close family members of Lgr5, have also been implicated in the regulation of stem cell properties. Lgr6 expression is found to mark hair follicle epidermal stem cells, whereas Lgr4 expression plays a major role in prostate stem cell function [79,105]. Lgr4 inactivation in mice leads to severe developmental deficiencies in multiple organs. Lgr4 knockout is associated with embryonic lethality in 60% of mice with surviving mice displaying infertility, delayed osteoblast differentiation, renal hypoplasia, malformation of the eye anterior segment, disrupted innate immunity and impaired mammary gland branching morphogenesis and mammary stem cells [106,107,108,109,110,111]. Lgr4 is also shown to enhance Wnt signaling through association with R-spondin, similar to the activity of Lgr5 [94]. These observations indicate a potential role for Lgr4 in regulating stem cells. Lgr4 is highly expressed during early stages of prostate development and its expression is restricted to the prostate stem cell (PSC) compartment in adults [79]. Deletion of Lgr4 expression leads to disrupted PSC cell fate determination resulting in arrested epithelial differentiation during prostate development [79]. These phenotypic effects are orchestrated by Lgr4-mediated potentiation of Wnt/β-catenin signaling. Consistently, Lgr4 deletion substantially reduces the intensity of β-catenin immunofluorescent staining localized to the nuclei of cultured prostate spheres [79]. Lgr4 signaling is enhanced by stimulation with Rspo3, and it is speculated that modest Wnt/β-catenin activity is required for PSC self-renewal and maintenance, while robust Wnt/β-catenin activity induced by combination of R-spondin and Lgr4 regulates PSC differentiation status [79]. Strikingly, the role of Lgr4 in cancer has been recently recognized, and Lgr4 has been reported to promote tumor metastasis through a PI3K-Akt-Erk-β-catenin-Tcf signaling axis in colon cancer [112]. Our unpublished data have also shown a fundamental role for Lgr4 in leukemia development through its regulation of LSC activity in AML [113]. These data indicate Lgr4 as a potential therapeutic target in cancer therapies.

**Figure 3:**
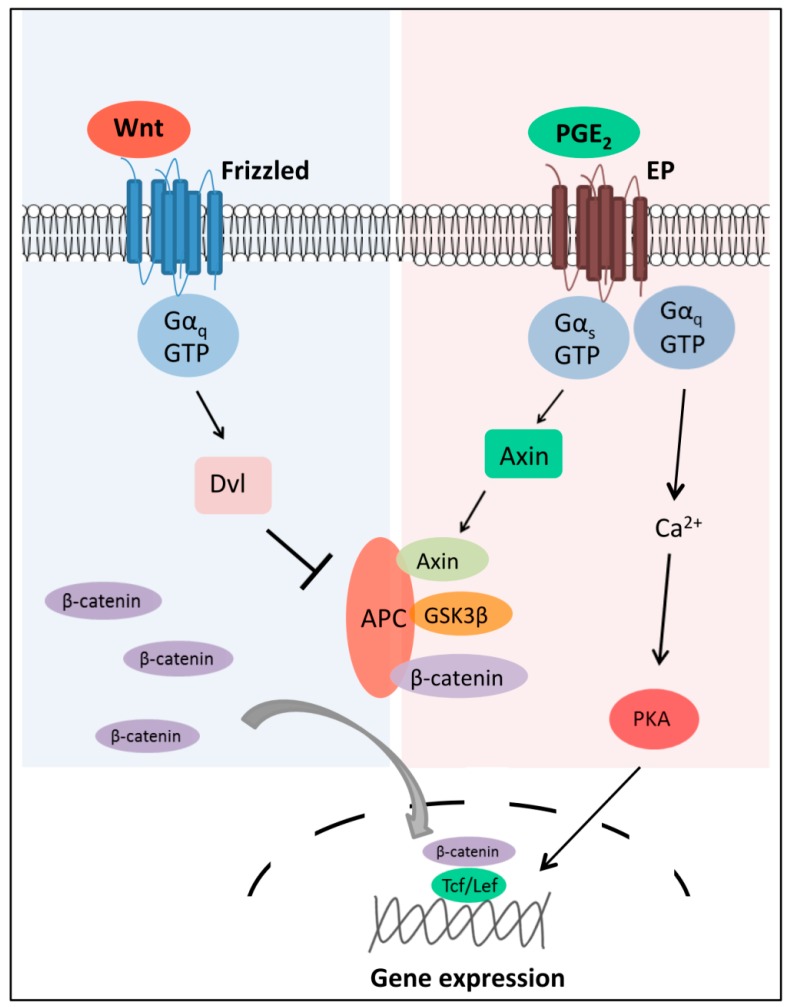
Diversity in GPCR-mediated regulation of Wnt/β-catenin. Wnt stimulation of the Fzd/β-catenin pathway activates the phosphoprotein Dvl, leading to inhibition of the β-catenin destruction complex composed of APC, the serine/threonine kinase glycogen synthase kinase 3β (GSK-3β) and Axin [116,117]. Cytoplasmic β-catenin then translocates to the nucleus, where it cooperates with the Tcf/Lef transcription factors to modify transcription of a set of Wnt target genes, primarily cell cycle regulators [53]. EP, a GPCR, utilizes distinct mechanisms to regulate β-catenin expression in cancer. EP promotes cancer cell growth by modulating a Ga_s_-Axin-β-catenin signaling axis [114]. Binding of the PGE_2_ agonist activates Gα_s_, which binds to Axin displacing APC from the destruction complex leading to stabilization and translocation of active β-catenin [56]. The diversity of GPCR signaling allows cancer cells to harness varying mechanisms to control key oncogenic targets. ↓: signaling activation; ⊥: signaling inhibition; grey curved arrow indicates translocation; dashed line represents nucleus.

The importance of GPCR signaling in driving LSC activity is further exemplified by the prostaglandin E (EP) receptors, another rhodospin class of GPCRs. Prostaglandins are a product of the cyclooxygenases Cox1 and Cox2 and their functions are initiated following binding of prostaglandins to their cognate GPCR receptors (EP1-4). EP1 is a Gα_q_-coupled receptor that promotes calcium mobilization and PKC activation, whereas EP2 and EP4 couple to Gα_s_ and stimulate cAMP accumulation and PKA activation [114]. We have previously shown that Cox1 and EP1 are upregulated in MLL fusion-derived LSCs, and that the Cox inhibitor, indomethacin, reduces β-catenin activity and impairs *in vivo* LSC function (*i.e.*, self-renewal and frequency) [78]. Although the mechanism of EP/Wnt signaling crosstalk in LSCs remains elusive, the complexity of such integrated signaling mechanisms has been interrogated in the case of colorectal cancers. Using colorectal carcinoma cells *in vitro*, it is shown that the EP ligand, PGE_2_, increases the activation of Tcf/Lef transcription factors, induces the loss of β-catenin phosphorylation and increases its nuclear accumulation [115]. This process is found to be independent of the PKA-cAMP pathway but rather dependent on the direct association of Gα_s_ with the β-catenin destruction complex member and Rgs protein, Axin [56]. Overexpression of the regulator of G protein (Rgs) binding domain of Axin, the region capable of stimulating GTPase activity and serving as a molecular scaffold, inhibits PGE_2_-induced transcriptional activation of Tcf/Lef [114]. Since the Rgs domain of Axin also serves as the site of APC binding, it is presumed that the binding of Gα_s_ to Axin results in the displacement of APC and loss of β-catenin phosphorylation leading to its nuclear accumulation [56] (Figure 3).

The EP receptors have demonstrated roles in a variety of malignancies and are widely expressed in primary invasive ductal carcinomas of the breast [118]. Emerging evidence indicates a role for EP4/Cox2 in maintaining the breast CSC phenotype. Kundu *et al.* [118] have observed upregulation of both EP4 and Cox2 in a sub-population of tumor-initiating cells and in metastatic and/or basal-type cells but not in non-metastatic or luminal-type cells, implicating association of EP4 and Cox2 with a malignant phenotype. As a result, a clinically relevant EP4 antagonist (RQ-15986) inhibits breast CSCs, metastasis and tumorigencity *in vivo* [118]. A number of clinical trials are currently being conducted to evaluate the effect of Cox2 inhibition in cancer but such a global targeting strategy is resulting in potential cardiovascular complications that may limit its applicability [42,119,120]. As such, EP4 antagonism likely represents a safer and more effective treatment strategy than global Cox2 inhibition [121].

### 2.4. The Complexity of GPCR-Mediated Signaling

The complexity of GPCR-mediated signaling is illustrated by its integrative crosstalk with non-GPCR driven signaling cascades, such as the EGFR (epidermal growth factor) pathway, as reported in many cell types [122,123,124,125,126]. EGFR controls a wide variety of biological processes such as proliferation, differentiation, migration and modulation of apoptosis [127]. Aberrant receptor signaling via overexpression, mutation or autocrine signaling loops has been frequently implicated in hyperproliferative disorders including cancer [128,129,130,131,132]. EGFR and its ligands are overexpressed and correlate with poor prognosis in various malignancies, including glioblastoma, breast, ovarian, gastric, esophageal and cervical cancers [132]. GPCR signaling is capable of transactivating the EFGR pathway and as such, a combination of the broad diversity of GPCR signaling with the potent signaling capacity of EFGR could serve as a paradigm for inter-receptor crosstalk [124].

**Figure 4:**
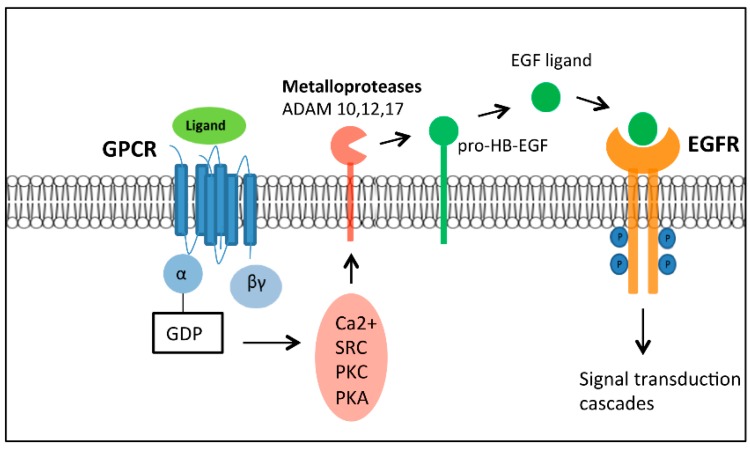
GPCR-mediated transactivation of EGFR. GPCRs induce the transactivation of EGFR through several mediators, including SRC kinases, Ca^2+^, PKC and PKA [133]. GPCR stimulation leads to activation of several members of the ADAM family of metalloproteases that generates the mature EGFR ligand from pro-HB-EGF. The release of the mature growth factor activates the EFGR and its subsequent downstream signaling cascades including activation of the mitogen-activated protein kinase (MAPK) transduction pathway controlling cell proliferation [135]. Adapted from [141].

EGFR transactivaton can proceed via several mechanisms (e.g., stimulation of GPCR) leading to activation of a metalloprotease, inducing pro-HB-EGF processing [133]. Subsequent release of the mature growth factor activates EGFR and its downstream signaling cascades (Figure 4) [133]. Metalloproteases are zinc dependent endopeptidases that elicit proteolytic degradation or activation of cell surface and extracellular matrix (ECM) proteins to modulate both cell-cell and cell-ECM interactions to influence cell proliferation, differentiation and survival [134]. Recently, the metalloproteases, which play a crucial role in the ligand-dependent EGFR transactivation mechanism, have been identified as members of the ADAM (a disintegrin and metalloprotease) family of zinc-dependent metalloproteases [135]. The widespread occurrence of this signaling mechanism has been verified in many cancer cell types, for example, HB-EGF shedding and subsequent EGFR transactivation is mediated by ADAM10 and ADAM17 in lung carcinoma cells [135,136]. ADAM10 is also involved in the shedding of other receptors such as the HER2 receptor resulting in its constitutive activation [137] and overexpression of ADAM10 has been reported in several malignancies including gastric, prostate, liver and breast cancer [138,139,140].

Despite the widespread overexpression of EGFR in cancer, EGFR-targeted therapies have produced only modest clinical responses in patients [142]. The diversity of GPCR, its heterogeneous expression in cancer and its complex cross signaling via GPCR-mediated EGFR-transactivation as discussed in this review may help explain this suboptimal clinical response. The progression of colon, lung, breast, head and neck, prostate and ovarian cancers have all been reported to be mediated, at least in part, by GPCR-EGFR crosstalk [143], indicating that combined GPCR and EGFR inhibition could induce more pronounced clinical responses. In support of this rationale, preclinical studies have shown that combined inhibition of GPCR and EGFR pathways can induce synergistic growth inhibition in head and neck squamous cell carcinoma, non-small cell lung cancer and pancreatic cancer [144,145,146]. Increased understanding of the specific signaling pathways involved in EGFR transactivation by GPCRs will facilitate the identification of multi-component molecular targeting strategies that may produce more pronounced clinical responses in patients. Furthermore, given the convincing evidence that is now emerging detailing the vital role played by GPCR in driving the CSC phenotype (summarized in Table 1), it is likely that integrative signaling pathway crosstalk as discussed in this review also contributes to the complexity of CSC signaling and its investigation is significantly warranted.

## 3. Clinical Implications of GPCR-Mediated Regulation of CSCs

GPCRs represent the largest class of cell surface receptors and are currently targeted by approximately 30%–40% of marketed drugs [147]. Several alternative therapeutic targeting strategies exist including broad spectrum drugs which simultaneously and non-specifically antagonize multiple GPCRs, the use of chimeric molecules comprised of several GPCR agonists/antagonists and mechanisms to inhibit GPCR oligomerization [148,149,150,151]. Recently, a novel strategy involving the use of pepducins that functions as agonists or antagonists and targets the intracellular domain of GPCRs has been developed [152,153].

Emerging evidence that the crucial CSC population, which drives resistance to therapy and patient relapse, is also reliant on GPCR-mediated signaling presents opportunities for therapeutic exploitation. Further characterization of key GPCR-mediated signaling cascades (e.g., Wnt/β-catenin) that contribute to CSC self-renewal will facilitate the development of CSC-targeted therapeutic strategies. Given the important role of aberrant Wnt/β-catenin in promoting CSC activity, substantial efforts have focused on the development of therapeutic approaches to target this pathway. However, progress has been thwarted by the extremely complex nature of Wnt/β-catenin signaling, notably the crosstalk with various non-Wnt factors [154,155]. The role of integrative signaling between GPCR- and non-GPCR-mediated pathways is beginning to be realized in cancer [124,141] and such complex signaling mechanisms must also be elucidated for the highly tumorigenic CSC population. This review has highlighted several GPCR-mediated CSC-targeting small molecule inhibitors that have shown promise in pre-clinical studies. Further investigations will hopefully drive preclinically validated inhibitors into clinical studies where their true therapeutic efficacies against the critical CSC population can be evaluated and the results of such studies will be eagerly anticipated.

## Figures and Tables

**Table 1:** The roles of GPCRs in normal and malignant stem cells. The roles of selected GPCRs in functional regulation of both normal and malignant stem cells are listed above. Abbreviations: ESC, embryonic stem cell; iPSC, induced pluripotent stem cells; AML, acute myeloid leukemia; LSC, leukemic stem cell; CSC, cancer stem cell; GPR84, G protein-coupled receptor 84; Lgr, leucine-rich repeat-containing G protein-coupled receptor; EP, prostaglandin E.

GPCR	Role	Stem Cell Type	Reference
Gα_s_	Promotes proliferation and pluripotency	Mouse ESCs	[11]
Gα_i_	Regulates morphology and cellular organization	Human iPSCs	[10]
Gα_i/o_	Regulates Wnt signaling activation	Human ESCs	[42,44]
Fzd7	Inhibition of Fzd7 induces significant morphological alterations with loss of pluripotency gene Oct4	Human ECSs	[57]
GPR84	Promotes β-catenin signaling and LSC maintenance	AML LSCs	[70]
Gα_q_	Enhances β-catenin signaling contributing to maintenance of fully-developed AML	AML LSCs	[71]
Lgr5	Potentiates Wnt signaling, drives migration and metastasis	Colon CSCs	[81,86,88]
Lgr6	Drives stem cell self-renewal	Hair follicle epidermal stem cells	[66]
Lgr4	Enhances Wnt signaling, promotes CSC self-renewal and maintenance	Prostate CSCs, mammary CSCs and AML LSCs	[66,92,98,100]
EP1	Regulates β-catenin driven self-renewal and stem cell frequency	AML LSCs	[65,101]
EP4	Enhances metastasis and tumorigenicity	Breast CSCs	[103]
